# Myeloperoxidase-Derived 2-Chlorohexadecanal Is Generated in Mouse Heart during Endotoxemia and Induces Modification of Distinct Cardiomyocyte Protein Subsets In Vitro

**DOI:** 10.3390/ijms21239235

**Published:** 2020-12-03

**Authors:** Jürgen Prasch, Eva Bernhart, Helga Reicher, Manfred Kollroser, Gerald N. Rechberger, Chintan N. Koyani, Christopher Trummer, Lavinia Rech, Peter P. Rainer, Astrid Hammer, Ernst Malle, Wolfgang Sattler

**Affiliations:** 1 Division of Molecular Biology and Biochemistry, Gottfried Schatz Research Center, Medical University of Graz, 8010 Graz, Austria; j.prasch@medunigraz.at (J.P.); eva.bernhart@medunigraz.at (E.B.); helga.reicher@medunigraz.at (H.R.); cnkoyani@yahoo.com (C.N.K.); christopher.trummer@medunigraz.at (C.T.); ernst.malle@medunigraz.at (E.M.); 2 Forensic Medicine, Medical University of Graz, 8010 Graz, Austria; manfred.kollroser@medunigraz.at; 3 Institute of Molecular Biosciences, University of Graz, 8010 Graz, Austria; gerald.rechberger@uni-graz.at; 4 Center for Explorative Lipidomics, BioTechMed Graz, 8010 Graz, Austria; 5 Department of Internal Medicine, Division of Cardiology, Medical University of Graz, 8010 Graz, Austria; cara.rech@medunigraz.at (L.R.); peter.rainer@medunigraz.at (P.P.R.); 6 Division of Cell Biology, Histology and Embryology, Gottfried Schatz Research Center, Medical University of Graz, 8010 Graz, Austria; astrid.hammer@medunigraz.at

**Keywords:** click chemistry, electrophile damage, fatty acids, hypochlorous acid, myeloperoxidase, proteomics

## Abstract

Sepsis is a major cause of mortality in critically ill patients and associated with cardiac dysfunction, a complication linked to immunological and metabolic aberrations. Cardiac neutrophil infiltration and subsequent release of myeloperoxidase (MPO) leads to the formation of the oxidant hypochlorous acid (HOCl) that is able to chemically modify plasmalogens (ether-phospholipids) abundantly present in the heart. This reaction gives rise to the formation of reactive lipid species including aldehydes and chlorinated fatty acids. During the present study, we tested whether endotoxemia increases MPO-dependent lipid oxidation/modification in the mouse heart. In hearts of lipopolysaccharide-injected mice, we observed significantly higher infiltration of MPO-positive cells, increased fatty acid content, and formation of 2-chlorohexadecanal (2-ClHDA), an MPO-derived plasmalogen modification product. Using murine HL-1 cardiomyocytes as in vitro model, we show that exogenously added HOCl attacks the cellular plasmalogen pool and gives rise to the formation of 2-ClHDA. Addition of 2-ClHDA to HL-1 cardiomyocytes resulted in conversion to 2-chlorohexadecanoic acid and 2-chlorohexadecanol, indicating fatty aldehyde dehydrogenase-mediated redox metabolism. However, a recovery of only 40% indicated the formation of non-extractable (protein) adducts. To identify protein targets, we used a clickable alkynyl analog, 2-chlorohexadec-15-yn-1-al (2-ClHDyA). After Huisgen 1,3-dipolar cycloaddition of 5-tetramethylrhodamine azide (N_3_-TAMRA) and two dimensional-gel electrophoresis (2D-GE), we were able to identify 51 proteins that form adducts with 2-ClHDyA. Gene ontology enrichment analyses revealed an overrepresentation of heat shock and chaperone, energy metabolism, and cytoskeletal proteins as major targets. Our observations in a murine endotoxemia model demonstrate formation of HOCl-modified lipids in the heart, while pathway analysis in vitro revealed that the chlorinated aldehyde targets specific protein subsets, which are central to cardiac function.

## 1. Introduction

Sepsis, a systemic inflammatory response that follows bacterial infection, is characterized by hypotension, ischemia, and multiple organ failure [1]. Cardiac dysfunction is a consequence of sepsis [2] and characterized by impaired contractility, diastolic dysfunction, and reduced ejection fraction [3]. The mechanisms underlying heart failure in acute sepsis are not entirely clear but include both inflammatory and metabolic alterations. Under septic conditions chemokine-, cytokine-, and tumor necrosis factor α (TNFα) release, alterations in nitric oxide (NO) production, dysfunctional Ca^2+^ homeostasis, activation of the complement and coagulation system, and impaired beta-adrenergic signaling contribute to organ dysfunction [4]. As an additional culprit, myocardial metabolism shifts from fatty acid (FA) and glucose oxidation toward aerobic glycolysis and lactate production [5].

In sepsis, lipopolysaccharide (LPS) binding to Toll-like receptor 4 (TLR4) induces NF-κB signaling, which induces auto-amplification of cytokine production (a process also termed ‘cytokine storm’; [6]). Increased levels of inflammatory cytokines further stimulate immunologically competent cells, generating a hyper-inflammatory environment that can induce organ damage/failure. Neutrophils are the first responders to pathogens and are rapidly recruited to sites of injury to remove invading pathogens. During activation, the content of specific neutrophil granules is released into vesicles formed around engulfed particles which are termed phagosomes. Within phagosomes, internalized microbes and/or fungi are surrounded by an unstirred water layer that contains high concentrations of granule enzymes [7]. During the early phase of phagocytosis, NADPH oxidase is assembled and generates superoxide anion radicals O_2_^-^, which are subsequently dismutated to H_2_O_2_, that together with chloride ions (Cl^−^), serve as substrate for myeloperoxidase (MPO)-mediated HOCl generation [8]. Following activation, neutrophils undergo apoptosis, a process preceded by the release of a network of extracellular DNA that contains MPO, elastase, cathepsin G, and other antimicrobial proteins [9]. These networks are termed neutrophil extracellular traps (NETs) and can kill microbes independently of phagocytosis.

Therefore the primary response mechanisms of neutrophils towards invading pathogens include receptor-mediated phagocytosis, intracellular killing, release of anti-microbial granule contents in a highly regulated fashion [10], and neutrophil extracellular trap (NET) formation [11]. Both, phagosomes and NETs contain ample amounts of the heme protein myeloperoxidase (MPO), which accounts for up to 5% and 1% of total cell protein content in neutrophils and monocytes, respectively [8]. Under physiological conditions, MPO is part of the innate immune system [8], while under chronic inflammatory conditions, MPO is considered as a disease modifier [12].

In the presence of chloride ions (Cl^−^; approx. 70 mM within phagosomes [8]), MPO converts the relatively weak two-electron oxidant H_2_O_2_ to highly reactive hypochlorous acid (HOCl), a prototypic example for a ‘reactive oxygen species (ROS) toxifier’ reaction. Chronic activation of phagocytes results in elevated levels of HOCl that can modify a range of biomolecules including antioxidants, nucleotides, DNA, (lipo)proteins, and lipids. Kalyanaraman and Sohnle demonstrated that opsonized zymosan-activated neutrophils at 5 × 10^6^ cells/mL generate approx. 90 μM HOCl [13]. Based on the HOCl production capacity of PMNs, it was calculated that HOCl concentrations could be as high as 340 µM under inflammatory conditions [14]. Thus, prolonged production of HOCl can cause tissue injury [15,16], MPO-derived oxidants contribute to atherosclerosis and plaque instability [17,18,19], attenuate diseases with a neuroinflammatory component [20], can lead to the formation of a chlorinated lipidome [16], or induce cardiac dysfunction [21]. A second heme peroxidase that was originally implicated in (cardio)vascular HOCl generation is Peroxidasin homolog (PXDN; formerly designated vascular peroxidase or cardiac peroxidase [22]). However, Paumann-Page and colleagues subsequently demonstrated that PXDN generates (pseudo)hypohalous acids from bromide, iodide, and thiocyanate, but not from chloride [23].

Plasmalogens, a class of ether-phospholipids that are particularly abundant in brain and heart, represent in vivo targets for MPO-mediated oxidative attack. During this oxidative modification reaction, the vinyl-ether bond at *sn1* is targeted by HOCl (generated by the MPO-hydrogen peroxide-chloride system) and gives rise to the formation of a lysophospholipid and a chlorinated aldehyde, with 2-chlorohexadecanal (2-ClHDA) as the prototypic representative originally identified by the Ford group [24]. 2-ClHDA is generated by activated neutrophils [25,26] and monocytes [27], and is present in human atherosclerotic lesions [18] and ischemic/reperfused myocardium [21]. This chloro fatty aldehyde is a potent neutrophil chemoattractant [26] and a potent inhibitor of vasculoprotective NO synthase in endothelial cells [25].

The electrophile 2-ClHDA impairs protein function by covalent modification, thereby triggering cytotoxic and adaptive responses that are typically associated with oxidative stress [28]. Consequently, conversion of (reactive) aldehydes to their corresponding alcohol and/or carboxylic acid analogues via the fatty alcohol cycle was considered as a protective pathway [29]. During this metabolic route, 2-ClHDA is oxidized to 2-chlorohexadecanoic acid (2-ClHA) [30]. However, also 2-ClHA may act as a lipotoxic compound and induces apoptosis, ROS formation, and ER stress in neutrophils [31] and brain endothelial cells [32]. Both, 2-ClHDA and 2-ClHA induce a potent inflammatory response in vitro and in vivo [33] and initiate NET formation without neutrophil activation and degranulation [34]. In patients with sepsis, the levels of circulating chlorinated FAs are significantly associated with acute respiratory distress syndrome and mortality [35].

During the present study, we sought evidence for MPO-mediated plasmalogen modification in an LPS-induced murine endotoxemia model that reflects some characteristic features observed in murine sepsis models [36]. For pathway analysis, we used the murine HL-1 cardiomyocyte cell line (further referred to as HL-1 cells). In this in vitro model, we characterized the formation of 2-ClHDA in response to exogenously added HOCl and conversion of 2-ClHDA to 2-ClHA. Using a clickable alkyne analog of 2-ClHDA, we identified protein targets in HL-1 cells by two-dimensional gel electrophoresis and tryptic peptide mapping by liquid chromatography-tandem mass spectrometry (LC-MS/MS).

## 2. Results

**Figure 1:**
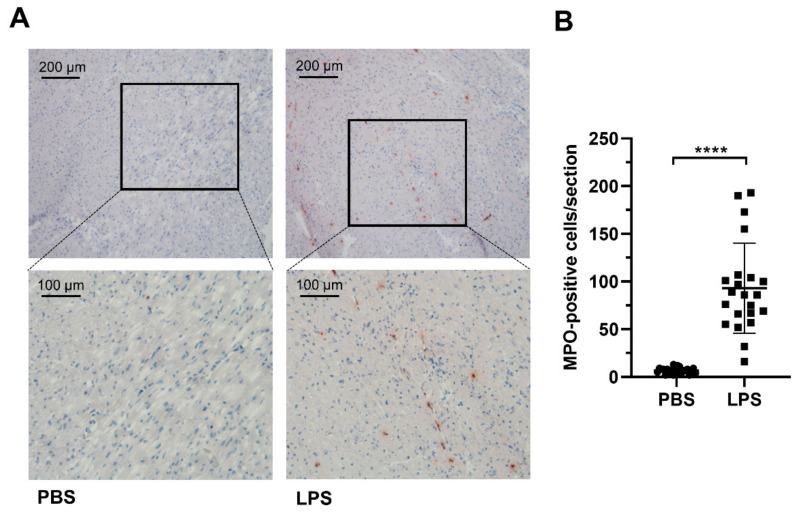
Immunohistochemical analysis of MPO expression in hearts of PBS- and LPS-treated mice. C57BL/6 mice received a single i.p. injection of PBS (200 µL) or LPS in PBS (from *Escherichia coli*, 0111:B4 in PBS, 8 µg/g body weight) and were sacrificed 12 h after the injection. (**A**) Representative MPO-immunostainings of hearts isolated from PBS- and LPS-injected animals are shown at low and high magnification. (**B**) Statistical evaluation of MPO-positive cells in the sections of the hearts of PBS- or LPS-injected mice. Cryosections of eight different heart regions from PBS- or LPS-injected animals (*n* = 3) were counted manually for MPO-positive cells. Lines indicate mean ± SD values. Unpaired student’s *t*-test; **** *p* ≤ 0.0001.

After 12 h of LPS administration (8 µg/g body weight, i.p.) to C57BL/6J mice, we observed a significant increase in myocardial MPO-positive cells when compared to sham (phosphate-buffered saline (PBS)-injected animals). In Figure 1A, immunohistochemical analysis of representative sections from ventricles isolated from PBS- and LPS-treated animals is shown at low and high magnification. Statistical evaluation of MPO-positive cells in the hearts isolated from PBS- or LPS-injected animals is shown in Figure 1B. These data demonstrate that the number of MPO-positive cells significantly increased from 6.7 ± 0.7 (PBS) to 92.9 ± 10.1 positive cells/field in the LPS-injected mouse cohort.

**Figure 2:**
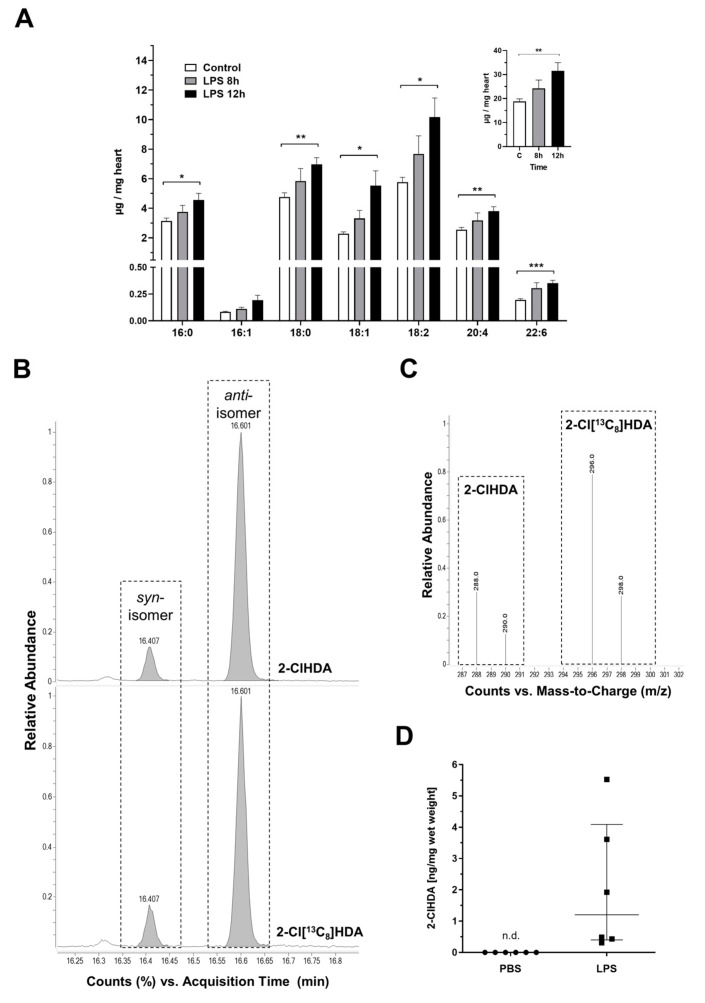
Accumulation of various fatty acid (FA) species and 2-ClHDA in LPS-treated hearts. C57BL/6 mice received a single i.p. injection of PBS (200 µ) or LPS in PBS (from *Escherichia coli*, 0111:B4 in PBS, 8 µg/g body weight) and were sacrificed 8 or 12 h after the injection. (**A**) Cardiac FA composition of PBS- and LPS-injected mice was analyzed by gas chromatography. Inset shows total FA concentrations. Data represent mean + SEM; * *p* ≤ 0.05; ** *p* ≤ 0.01; *** *p* ≤ 0.001; unpaired student’s *t*-test (*n* = 5–6). (**B**–**D**) Cardiac 2-ClHDA concentrations were quantified by selected ion monitoring (SIM) NICI–GC–MS analysis using 2-Cl[^13^C_8_]HDA as internal standard. (**B**) SIM chromatograms of a representative cardiac lipid sample (top; 12 h post LPS treatment) and the synthetic standard (bottom). Boxed areas indicate the elution profiles of the syn- and anti-PFB-oxime derivatives of 2-ClHDA and 2-Cl[^13^C_8_]HDA. (**C**) Fragment ion intensity ratios of 2-ClHDA (*m/z* = 288, 290) and the internal standard (*m/z* = 296, 298) of the peaks highlighted in (**B**). (**D**) Formation of 2-ClHDA in the hearts of LPS-treated animals (*n* = 6) measured 12 h after a single systemic LPS injection. Lines represent median with interquartile range, unpaired student‘s *t*-test, non-detectable (n.d.).

Generation of ROS during endotoxemia was proposed as one of the mechanisms contributing to myocardial dysfunction via TLR4-mediated pathways [37]. Since the content of polyunsaturated FAs is considered a measure for oxidative stress, we compared the cardiac FA composition of PBS- and LPS-injected animals (Figure 2A). These analyses revealed FA content of 18.7 µg/mg wet tissue in controls, while endotoxemia increased total FA concentrations to 23.9 and 31.7 µg/mg wet tissue (8 and 12 h post LPS, respectively; inset in Figure 2A). In terms of individual FA species, the quantitatively most pronounced increase was observed for C18:2, followed by C18:1, C18:0, C16:0, and C20:4 (Figure 2A). These results indicate profound changes in cardiac FA/lipid utilization during sepsis [3] rather than designating general oxidative stress conditions.

We then aimed to characterize MPO-dependent lipid modification in the hearts obtained from LPS injected mice. To determine whether 2-ClHDA is generated in response to endotoxemia, cardiac lipid extracts were converted to the corresponding pentafluorobenzyl (PFB) derivatives and analyzed by negative ion chemical ionization-gas chromatography-mass spectrometry (NICI-GC-MS). The retention times and the mass spectrum of the identified product were identical to the PFB-derivative of a synthetic 2-ClHDA standard (Figure 2B,C). The fragment ions at *m/z* 288/290 are detected in a 3/1 ratio indicating a mono-chlorinated analyte. Using a stable isotope labeled internal standard (2-Cl [^13^C_8_] HDA), we quantitated accumulation of 2-ClHDA in the hearts of LPS-exposed animals (Figure 2D). As can be seen from the scatterplots, 2-ClHDA concentrations in the hearts of PBS-injected mice were below the limit of detection (31 ng on column). In contrast, 2-ClHDA was detectable in the hearts of LPS-treated mice, albeit the concentrations were variable within a range between 0.5 and 5.8 ng/mg wet tissue.

**Figure 3:**
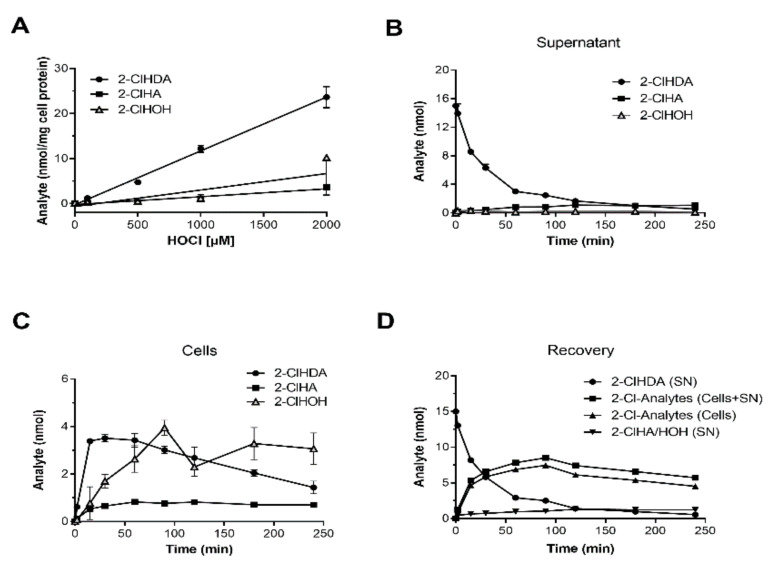
In vitro formation and metabolism of 2-ClHDA in the murine HL-1 cardiomyocyte cell line. HL-1 cells were incubated with increasing concentrations of NaOCl or 2-ClHDA (15 µM). (**A**) After treatment with indicated concentrations of NaOCl for 1 h, cells were extracted in the presence of the corresponding internal standard as outlined in Materials and Methods. After conversion to their corresponding PFB-derivatives, 2-ClHDA, 2-ClHA, and 2-ClHOH concentrations were quantitated by NICI-GC–MS analysis. Results are displayed as mean ± SD (*n* = 3). (**B**,**C**) Cells were incubated with 15 µM 2-ClHDA for up to 4 h. At the indicated time points, 2-ClHDA, 2-ClHA, and 2-ClHOH concentrations were analyzed by NICI-GC–MS analysis in (**B**) the cellular supernatants and (**C**) HL-1 cells. Data represent mean ± SD values (*n* = 3). (**D**) Time-dependent recovery of 2-Cl-metabolites in HL-1 cells. Data represent loss of 2-ClHDA from the supernatant (SN), recovery of 2-Cl-Analytes (sum of 2-ClHDA, 2-ClHA, and 2-ClHOH in the SN or cells), and recovery of 2-ClHA plus 2-ClHOH in the supernatant (for reasons of clarity only mean values are shown).

The next set of experiments aimed to investigate whether 2-ClHDA formation can be mimicked in vitro and to determine reagent hypochlorite (NaOCl) concentrations that induce plasmalogen modification in HL-1 cells. NICI-GC-MS analyses of lipid extracts from NaOCl-treated HL-1 cells revealed a linear increase of cellular 2-ClHDA concentrations in response to increasing oxidant concentrations (Figure 3A). Linear regression analysis revealed that approx. 1.2 × 10^−2^ nmol 2-ClHDA/mg cell protein is formed per nmol NaOCl. The corresponding numbers for 2-ClHA and 2-chlorohexadecanol (2-ClHOH) were 1.7 × 10^−3^ and 3.6 × 10^−3^ nmol/mg cell protein/nmol HOCl (Figure 3A). The formation of these analytes is most likely due to downstream metabolism of newly generated 2-ClHDA by fatty aldehyde dehydrogenase (FALDH) [29,30].

To study this aspect in more detail, HL-1 cells were incubated with 2-ClHDA (15 µM; 15 nmol/well) in a time-dependent manner and resulting concentrations of 2-ClHDA, 2-ClHA, and 2-ClHOH were quantitated in lipid extracts from supernatant and cells. In the supernatant, 2-ClHDA concentrations time-dependently decreased (from 15 to 1.6 nmol at 240 min) with a τ/2 of 20.6 min (acc. to 2-ClHDA_t_ = 2-ClHDA_0_ × e^−0.03365 × t^). This decrease was accompanied by the release of 2-ClHA (1.07 nmol) and 2-ClHOH (0.13 nmol) into the medium (Figure 3B). In the cellular lipid extracts, 2-ClHDA increased up to 30 min (3.5 nmol) and then decreased in a linear fashion. 2-ClHA remained constant (0.8 nmol) over the entire incubation period. 2-ClHOH concentrations reached maximum levels at 90 min (3.7 nmol; Figure 3C). Total recovery from cells and medium is shown in Figure 3D. These data indicate that 39% of originally added 2-ClHDA was recovered as extractable metabolites (5.8 nmol), suggesting that 61% of 2-ClHDA is lost. In addition to glutathione (GSH) [38] and phosphatidylethanolamine [39], proteins are targeted by MPO-derived oxidants [40], a process that was termed ‘alkylation damage’ [41].

**Figure 4:**
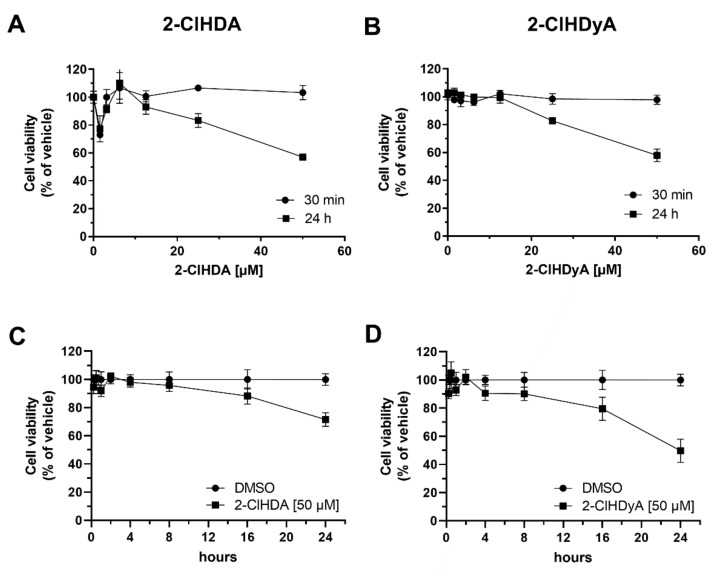
Effects of 2-ClHDA and 2-ClHDyA on cell viability. Viability of HL-1 cells was assessed with the CellTiter-Glo 3D kit detecting ATP as an indicator of cell viability. Cells were treated with (**A**) 2-ClHDA or (**B**) 2-ClHDyA at indicated concentrations for 30 min and 24 h. The time-dependent effect of 50 µM 2-ClHDA and 2-ClHDyA on cell viability is shown in (**C**) and (**D**), respectively. DMSO was used as vehicle control. Data represent mean ± SD values (*n* = 3).

To get an indication which cardiomyocyte proteins form adducts 2-ClHDA, we chose a Huisgen 1.3-dipolar cycloaddition (‘click chemistry’) approach. Covalent modification of proteins by 2-ClHDA occurs via the functional aldehyde or chlorine group that can attack amino acid side chains of lysine, cysteine, histidine, and arginine. During these experiments, HL-1 cells were exposed to a 2-ClHDA-bioortholog containing a terminal alkyne group at C_15_ (2-chlorohexadec-15-yn-1-al, termed 2-ClHDyA). Electrophiles containing a terminal alkyne (or azide) group are accessible to Cu^2+^-catalyzed Huisgen 1.3-dipolar cycloaddition, allowing conjugation of a reporter molecule. During the present study, 5-tetramethylrhodamin azide (N_3_-TAMRA) was used as reporter fluorophore. To perform these studies at non-toxic 2-ClHDyA concentrations, HL-1 cells were incubated with 2-ClHD(y)A and ATP (as a measure of intact cellular energy metabolism) was quantitated using the CelltiterGlo assay. Concentration dependent experiments (2.5–50 µM of 2-ClHDA or 2-ClHDyA) revealed that neither the parent compound nor the alkyne derivative impact cellular ATP levels at 30 min (Figure 4A,B). In contrast, ATP levels were reduced by approx. 40% after 24 h (Figure 4A,B; both compounds 50 µM). Time-dependent experiments using vehicle (dimethyl sulfoxide (DMSO)), or 50 µM 2ClHDA (Figure 4C) or 2-ClHDyA (Figure 4D) revealed that ATP levels (and thus cell viability) start to decrease at incubation times > 4 h.

**Figure 5:**
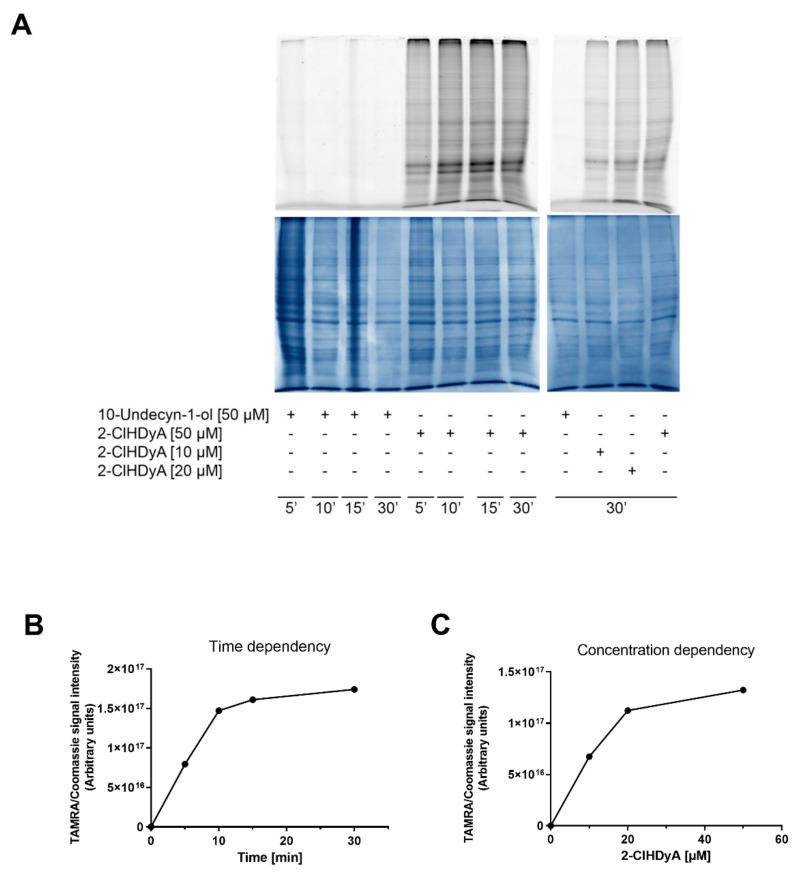
Adduct formation of 2-ClHDyA with cardiac proteins. (**A**) HL-1 cells were incubated in the absence (‘-‘) or presence (‘+’) of 50 µM 2-ClHDyA for the indicated time points (left panel) or with 10, 20, and 50 µM 2-ClHDyA for 30 min (right panel). 10-Undecyn-1-ol served as a negative control. Cell lysates were subjected to click chemistry with N_3_-TAMRA, separated by SDS-PAGE, and imaged using a Typhoon 9400 scanner (upper panel). Coomassie Brilliant Blue staining was performed to verify equal protein loading (lower panel). (**B**) Time- and (**C**) concentration-dependent increase in fluorescence intensities normalized to overall protein intensity/lane from the gels shown in (**A**). One representative experiment is shown.

To establish optimal click conditions, HL-1 cells were incubated in the presence of 10-undec-yn-ol (a negative control containing a terminal alkyne residue but lacking a reactive functional head- or side-group capable of protein modification) and 2-ClHDyA (10, 20, and 50 µM) for the indicated times. Thereafter, cells were lysed, clicked with N_3_-TAMRA, and proteins were separated by 1D-PAGE. 2-ClHDyA-modified proteins (containing the TAMRA fluorophore) were visualized by fluorescence scanning (ex 532/em 580 nm). As expected, no fluorescent signal was detected for 10-undecyn-1-ol. In the respective lanes of 2-ClHDyA-treated protein lysates (5–30 min), a time- and concentration-dependent increase in fluorescence signal intensity was observed (Figure 5A; upper panel). Subsequently, the gels were stained with Coomassie Brilliant-Blue (lower panel) and fluorescence intensities were normalized to the corresponding Coomassie stained lanes. This normalization revealed that protein labeling was near saturation at 20 µM 2-ClHDyA after 15 min (Figure 5B) and reached a plateau after 30 min at 50 µM 2-ClHDyA (Figure 5C).

Thus, for 2D-GE experiments, HL-1 cells were exposed to 50 µM 2-ClHDyA for 30 min (non-toxic conditions; Figure 4) prior to N_3_-TAMRA clicking. Following click chemistry, cell lysates were separated by 2D-GE and subjected to fluorescence scanning. Fluorescent spots from three individual 2D-gels were picked, tryptically digested, and identified by LC-MS/MS. 90 spots contained keratin and were considered contaminations by dust or human skin. Of the remaining 81 protein hits, 51 were identified with a MS/MS score >18 in two gels and are listed in Table 1 along with SwissProt accession number, theoretical molecular mass, and pI. Proteins are grouped according to pathway assignment and within the groups, the corresponding proteins are listed alphabetically. These groups contain:

(i). Cytoskeletal proteins that belong to microfilaments (Actbl2), intermediate filaments (e.g., Des and Vim), or microtubules (members of the tubulin beta chain family);
(ii). Chaperones and stress response including heat shock proteins (HSPs), Cct subunits of the chaperonin-containing T-complex (CCT), protein disulfide isomerases (Pdia3 and 6), and eukaryotic translation initiation factors;
(iii). Proteins involved in cellular energy metabolism, primarily cytosolic glycolytic enzymes but also mitochondrial proteins like Idh3a, Ndufs2, Uacrc1 and 2; and
(iv). Miscellaneous proteins that were not included in the groups listed above.

**Figure 6:**
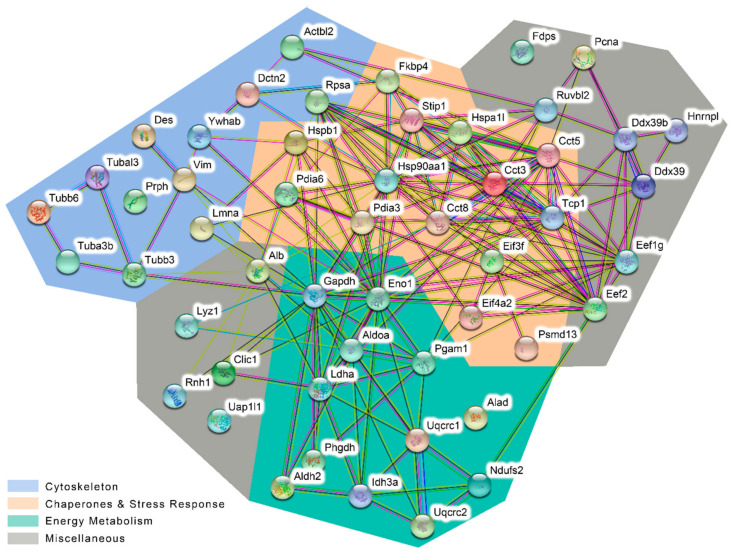
Specific network analysis of HL-1 proteins modified by 2-ClHDyA.

To get an indication about functional interactions between targeted proteins and their involvement in specific biological processes, STRING analysis was performed. A graphical presentation of interactions between the identified proteins is shown in Figure 6. Proteins present in this STRING network were broadly classified into three functional and interlinked categories, namely, cytoskeleton (blue), chaperones and stress response (orange), and energy metabolism (green), indicating that 2-ClHDA targets functional and interdependent protein systems in HL-1 cells. Proteins that were not grouped in either category are shown in the grey areas.

Cells were treated with 50 µM 2-ClHDyA for 30 min and cell lysates were subjected to click chemistry, 2D-GE and LC-MS/MS analysis. STRING analysis of the 51 identified proteins (full names in Table 1) revealed their involvement in three biological processes: cytoskeleton (blue), chaperones and stress response (orange), and energy metabolism (green). Proteins not fitting in either group are shown in grey areas.

Gene ontology (GO) enrichment analysis of biological processes identified enrichment of protein data sets in subgroups including protein folding and diverse subsets related to energy metabolism (Table S1). In line, Kyoto Encyclopedia of Genes and Genomes (KEGG) pathway analysis revealed significant enrichment for glycolysis/gluconeogenesis, amino acid biosynthesis, carbon metabolism, and metabolic processes (Table S2). In summary, these findings indicate that 2-ClHDyA covalently targets proteins that maintain cytoskeletal integrity, protein folding, and energy homeostasis in cardiomyocytes.

## 3. Discussion

Inflammatory and metabolic events are pivotal factors contributing to sepsis-associated cardiac dysfunction [3]. During the inflammatory response, enhanced adhesion molecule expression leads to neutrophil and monocyte infiltration in the heart and complement activation in conjunction with immune cell-derived mediators contribute to organ injury [42,43]. Neutrophil accumulation and activation is accompanied by the release of cytotoxic enzymes including neutrophil-derived elastase (NE) and MPO, resulting in local (oxidative) stress conditions. In fact, MPO is involved in cardiac remodeling and dysfunction through multiple pathways [44]. MPO-derived oxidants increase myocardial collagen deposition [45], play a major role in left ventricular remodeling after myocardial infarction (MI) [46], and increase vulnerability to atrial fibrillation [47]. MPO-mediated activation of MMP7 and subsequent connexin43 destruction was identified as an underlying mechanism causing arrhythmias and sudden death after MI [48]. The outcome of experiments performed during the present study is reminiscent of several of these manifestations. The major in vivo findings obtained during the present study are the accumulation of MPO-positive cells, profound changes in cardiac FA profiles, and HOCl-mediated plasmalogen modification resulting in formation of 2-ClHDA in the hearts of endotoxemic mice. Pathway analysis in HL-1 cells in vitro revealed the formation of 2-ClHDA in HOCl-exposed cells, redox metabolism of 2-ClHDA, and covalent protein adduct formation with the clickable alkyne derivative 2-ClHDyA.

In the murine endotoxemia model used during the present study, we observed accumulation of MPO-positive cells in the hearts of LPS-exposed mice (Figure 1). Initially, total FA concentration in cardiac lipid extracts of PBS- and LPS-injected animals were determined to get an indication of oxidative stress conditions that lead to polyunsaturated FA consumption [49]. However, here we observed significantly increased concentrations of most FA species in hearts of LPS-injected animals (Figure 2). These findings point towards reduced cardiac FA utilization as energy substrates in β-oxidation. In line, cardiometabolic deficits including reduced palmitate oxidation, increased triglyceride accumulation, and suppression of the PGC-1 pathway were observed in cardiomyocytes of LPS-injected mice, a metabolic phenotype reverted upon PGC-1β reactivation [50].

The Ford group presented the first experimental evidence for cardiac 2-ClHDA formation in rats subjected to left anterior descending coronary artery occlusion [21]. Subsequently, the presence of relatively long-lived chlorinated FAs was demonstrated in urine of LPS-exposed rats [51], Sendai-virus infected mice [30], and in plasma and several organs of a cecal slurry-induced rat sepsis model [52]. The same group reported that the concentrations of 2-chloropalmitic- and 2-chlorostearic acid concentrations strongly correlate to sepsis-associated acute respiratory distress syndrome and mortality [35]. The concentrations of cardiac 2-ClHDA observed here (Figure 2) are slightly lower as reported for a cecal slurry-induced rat sepsis model, most likely due to the lower MPO activity in mice [52]. Along this line, it might be important to note that the sequence identity between human and murine MPO is 86.5% (BLAST analysis in Uniprot, https://www.uniprot.org/; P05164 MPO_human vs. P11247 MPO_mouse).

Increasing evidence suggests that lipid-derived electrophiles have the potential to perform specific signaling tasks. This is achieved by modification of first line sensor proteins via thiols in Cys-residues that differ in nucleophilicity [53]. 2-ClHDA, a reactive electrophile species (RES), belongs to the α-halocarbonyl species [54] and participates in redox signaling [28,55]. Within this concept, downstream metabolism of reactive electrophiles is an important issue in terms of toxicological considerations [56]. In neutrophils and human coronary endothelial cells, 2-ClHDA is metabolized to 2-ClHA and 2-ClHOH [57] via FALDH-mediated pathways [30]. Both of these metabolites are still amenable to nucleophilic attack at C_2_ through chlorine abstraction [32], but lost their ability for Schiff’s base formation. In addition, 2-ClHA might display toxic potential by inducing NETosis [34]. Esterification of 2-ClHA into complex lipids [52,58] or catabolism by ω-oxidation and subsequent β-oxidation (starting at the ω-C-atom) [51] likely represent detoxification pathways that might be considered cardioprotective. In line, pharmacological activation of ALDH2 was shown to reduce ischemic damage to the heart in a rat model [59,60].

During the present study, we observed a half-life time of approx. 20 min for a bolus of 2-ClHDA (Figure 3) and recovered roughly 40% of originally added 2-ClHDA as 2-ClHA or 2-ClHOH from HL-1 cells. The remaining proportion was not amenable to direct PFB-derivatization under the analytical conditions employed here. This is most probably due to several overlapping pathways including esterification of 2-ClHA into complex lipids [58], and GSH- [38], lipid- [39], or protein-adduct formation [28].

To characterize protein targets for 2-ClHDA in HL-1 cells, a proteomic approach was performed. Using an alkyne analog of the parent compound, we identified 51 proteins (MS/MS score >18; Table 1) that were modified by 2-ClHDyA. To avoid the induction of lethal cellular injury, we chose non-toxic incubation conditions (Figure 4) and hypothesized to predominately observe adduct formation with high susceptibility protein targets. Most (>70%) of the identified protein candidates could be grouped into three distinct subsets, including chaperone and stress response proteins, metabolic enzymes, and cytoskeletal proteins. Of note, these protein families were shown to comprise the most sensitive systems toward low-level electrophile stress [41].

The observation of chaperone and stress response protein modification by 2-ClHDyA in HL-1 cells is intriguing and suggests that 2-ClHDA elicits a cellular defense response, as shown for other non-chlorine containing electrophiles [61]. Within the concept of redox signaling, growing evidence suggests that the modification of specific sensor proteins conveys information on the state of cellular homeostasis. RES are able to activate the cellular defense machinery, including the Keap1/Nrf2 antioxidant response [62], the unfolded protein response [32], or the heat shock response (HSR; [54]). Here, we show that 2-ClHDyA targets cytosolic and nuclear members of the HSR protein network (Table 1 and Figure 6). This is reminiscent of what has been reported for other prototypic RES like 15d-PGJ2, 4-hydroxynonenal (HNE), or acrolein that are potent inducers of the HSR [54]. Small HSPs are specifically enriched in the heart and HSPB1 (also identified during the present screen) was shown to take a central role in cardiac redox metabolism [63]. HSPB7—a member of the cardiovascular small HSP family [64]—acts as a kinetically privileged sensor of HNE with RES sensing being accomplished by a single reactive cysteine [65]. We have also identified three subunits (Cct-3, -5, and -8) of CCT, a central ATP-dependent chaperonin complex that folds cytosolic proteins and links the cytosolic chaperone machinery to the HSR by physical interaction with heat shock transcription factor 1 (HSF1) [66].

A second class of proteins identified as 2-ClHDyA adducts were enzymes involved in energy metabolism. Among these mainly glycolytic enzymes including AldoA, Phgdh, Pgk1, Pgam1, Eno1, and LDHa and LDHb were identified in TAMRA-tagged protein spots. From these results, it appears that glycolytic enzymes are overrepresented as 2-ClHDyA targets. This is most likely a reflection of the fact that HL-1 cells exhibit low capacity oxidative phosphorylation but cover their ATP demand mainly by a high rate of glycolysis, which is in contrast to primary cardiomyocytes that cover approx. 70 % of their ATP demand from β-oxidation [67]. 2-ClHDyA-modified proteins were also observed in mitochondria: In this compartment, Ndufs2, the core subunit of Complex I that transfers electrons from NADH to the respiratory chain, and two subunits of mitochondrial respiratory chain complex III (Uqcrc1 and Uqcrc2) that establish the proton gradient that ultimately fuels ATP production, were also subject to 2-ClHDyA adduction. In murine chagasic cardiomyopathy (elicited by the parasite *Trypanosoma cruzi*), oxidative adduct formation of mitochondrial complex subunits (including Ndufs2, Uqcrc1 and -2, as observed here) results in catalytic inactivation and a subsequent decrease in mitochondrial ATP synthesis in infected hearts [68].

Respiratory activity in mitochondria of cardiomyocytes depends on an intact cytoskeleton due to the formation of a multiprotein complex, termed the ‘mitochondrial interactome’ or ‘mitochondrial interactosome’. The central player in this model is voltage-dependent anion channel (VDAC), a transporter located in the outer mitochondrial membrane that regulates entry of respiratory substrates, ADP, and Pi [69]. At the same time, high-energy phosphates are shuttled out to enter energy transfer networks. During the present study, we observed 2-ClHDyA modification of microtubule- (Tuba3b, Tubal3, Tubb3, and Tubb6) and class III intermediate filament-associated proteins (Vim, Des, and Prph) that regulate VDAC permeability mitochondrial energy flux and thus cardiomyocyte function [69,70]. Vim plays an important role in correct positioning of mitochondria, maintenance of mitochondrial morphology, and mitochondrial function [71]. Immunofluorescence analyses demonstrated that Tubb2 is responsible for the structural and functional interactions of microtubules with VDAC in primary cardiomyocytes [72].

Our study has also some limitations: In the hearts of LPS-injected mice, only the primary plasmalogen modification product 2-ClHDA was quantitated. Downstream metabolites (2-ClHA or other chlorinated FAs that are implicated in sepsis and/or organ dysfunction; [35,52]) were not included in our analyses. On the other hand, the in vitro proteome study provides no information about kinetics and functional consequences of 2-ClHDyA adduct formation (i.e., rate of gain or loss of protein function). We are also not able to comment on target localization (exact position of the amino acid within the target protein sequence) and/or target occupancy (number of amino acids in the target protein that bound 2-ClHDyA).

Despite these limitations, our findings in a murine endotoxemia model demonstrate (i) the accumulation of MPO-positive cells in the mouse heart, (ii) aberrant cardiac FA utilization, and (iii) HOCl-mediated plasmalogen modification that gave rise to the formation of 2-ClHDA. Pathway analysis in HL-1 cells in vitro revealed the formation of 2-ClHDA in HOCl-exposed cells, redox metabolism of 2-ClHDA, and covalent protein adduct formation with the clickable bioortholog 2-ClHDyA. The outcome of our experiments is reminiscent of several pathways known to induce cardiomyocyte dysfunction in sepsis.

## 4. Materials and Methods

### 4.1. Animals

C57BL/6 mice (10–12 weeks, 20–30 g) were obtained from the Department of Laboratory Animal Science (Himberg, Austria). All animals were kept on a 12 h light/dark cycle with free access to food and water. Animal experiments were approved by the Austrian Federal Ministry of Science, Research, and Economy, Division of Genetic Engineering and Animal Experiments (BMWF-66.010/0067-V/3b/2018; issued on 7 June 2018). Animals were injected i.p. with PBS or LPS (from *Escherichia coli* 0111:B4, 8 µg in PBS/g body weight). After 4, 8, or 12 h (as indicated), mice were anesthetized with 150 µg/g pentobarbital and transcardially perfused with PBS. Time points were chosen to minimize animal suffering according to the 3R principles (in our experience animal survival at 12 h is 100%). Hearts were removed and frozen in liquid nitrogen until further processing.

### 4.2. Immunohistochemistry

Serial axial cryosections (5 µm) were collected on glass slides, air dried for 2 h at room temperature (RT), and frozen for further use. For experiments, the slides were thawed fixed with acetone for 5 min at RT. After rehydration in PBS and blocking with UV Ultra protein block (Thermo Scientific 10 min, RT), sections were stained with rabbit anti-human MPO antibody (DAKO, 1:500, 30 min, RT) and horseradish peroxidase (HRP)-labeled goat-anti-rabbit IgG (Biorad, Vienna, Austria; 1:200, 30 min, RT). After development with 3-amino-9-ethylcarbazole (Lab Vision AEC Substrate System) and counterstaining with Mayer’s hemalum solution, sections were mounted with Kaiser’s glycerol gelatin and visualized with a Leica DM600B microscope.

### 4.3. Cell Culture

HL-1 cells (a murine cardiomyocyte cell line, Sigma-Aldrich, Vienna, Austria) were cultured in fibronectin (0.5%)/gelatin (0.02%)-coated flasks and maintained in Claycomb medium (Sigma-Aldrich, St. Louis, MO, USA) containing 10% (*v*/*v*) fetal bovine serum (FBS, Thermo Fisher Scientific, Waltham, MA, USA), 0.1 mM norepinephrine, 2 mM L-glutamine, 100 IU/mL penicillin, and 100 μg/mL streptomycin (Sigma-Aldrich) [73] and kept at 37 °C under 5% CO_2_.

### 4.4. FA Analysis

Mouse hearts were homogenized in a Precellys homogenator in PBS. Lipids were extracted twice with chloroform/methanol (2/1, *v*/*v*), dried under a stream of nitrogen, and re-dissolved in toluene. After addition of the internal standard (pentadecanoic acid), lipids were trans-esterified (1.2 mL toluene and 1 mL boron trifluoride-methanol (20%)) at 110 °C for 1 h. GC analysis of FA methyl esters was performed as described [74] and concentrations were quantitated by peak area comparison with the internal standard and normalization to wet tissue weight.

### 4.5. Analysis of 2-ClHDA in Murine Cardiac Tissue

Mouse hearts were homogenized in a Precellys homogenator in distilled water. Prior to lipid extraction, the internal standard was added (1 µg 2-Cl[^13^C_8_]HDA). Lipids were extracted two times with chloroform/methanol (2/1, *v*/*v*) and the lower phase was dried under a stream of nitrogen. Lipid extracts from one heart were reconstituted in 100 μL hexane and fractionated on silica gel 60 plates using hexane/diethyl ether/acidic acid (50/50/1, *v*/*v*/*v*) as mobile phase. Fractions comigrating with an authentic 2-ClHDA standard were scraped off, extracted from the TLC sorbent twice with chloroform, converted to the corresponding PFB-oximes, and analyzed by GC–MS as described below.

### 4.6. Metabolic Conversion of 2-ClHDA to 2-ClHA and 2-ClHOH

HL-1 cells were treated on uncoated 6-well plates in serum-free medium in the presence of 2-ClHDA (15 µM) for indicated time-periods. Following treatment, supernatant was spiked with internal standards (Table 2) and extracted twice with 2 mL chloroform/methanol (3/1, *v*/*v*). Cell monolayers were extracted in hexane/isopropanol (3/2, *v*/*v*) and protein concentration was determined using the Lowry assay. Extracts were dried under a stream of nitrogen and stored at −20 °C until derivatization.

### 4.7. Derivatization Procedures

Preparation of PFB-oxime derivatives of 2-ClHDA was performed as previously described [75]. FAs were converted to the corresponding PFB-ester derivatives in 100 μL 0.35% (*v*/*v*) PFB-bromide in acetonitrile and 20 μL N,N-diisopropylethylamine for 30 min at RT. Fatty alcohols were converted to PFB-esters using 100 μL 0.4% (*v*/*v*) pentafluorobenzoyl chloride in acetonitrile for 1 h at 80 °C. Derivatization reagents were evaporated on an Eppendorf concentrator. Samples were redissolved in 100 μL toluene, transferred to autosampler vials, and stored at −20 °C until GC/MS analysis.

### 4.8. NICI-GC-MS Analysis

Samples were analyzed on an Agilent 7890B GC (helium was used as carrier gas, 2 mL/min) using a HP-5MS capillary column (30 m, 0.25 mm inner diameter, 0.25 μm phenyl methyl siloxane coating) and a 5977B mass spectrometer (Agilent). Injection volume was 1 µL. Injector temperature was set to 250 °C and ion source temperature was 310 °C. The oven temperature was maintained at 60 °C for 2.25 min, increased during the first ramping step at a rate of 20 °C/min to 175 °C, and held at 175 °C for 1 min. In the second ramping step, the temperature was raised at a rate of 15 °C/min to 280 °C and held at 280 °C for an additional 4 min. All spectra were monitored in NICI mode (methane was used as reagent gas), either in full scan or using selected ion monitoring mode (SIM). In SIM, target compounds were identified at molecule specific mass-to-charge ratios and characteristic isotope distribution of chlorine (Cl^35^/Cl^37^, 3/1). Quantitation was performed by peak area comparison with internal standards (Table 2).

### 4.9. HOCl-Induced Formation of 2-ClHDA and 2-ClHA in HL-1 Cardiomyocytes

NaOCl (0.1–2 mM) was added to HL-1 cells cultured on uncoated 6-well plates in serum-free medium. After 1 h, cells were washed, lipids were extracted twice (1 mL of hexane/isopropanol (3/2, *v*/*v*); orbital shaker, 30 min). Cells were lysed in 500 µL 0.3 M NaOH (orbital shaker, 1 h at RT). Protein content was determined using the Lowry assay. Lysates were spiked with corresponding internal standards (Table 2), extracted (chloroform/methanol, 2/1, *v*/*v*), converted to PFB-oxime and PFB-ester derivatives, and analyzed by NICI-GC-MS as described above.

### 4.10. Synthesis and Analysis of Chlorinated Lipids

2-ClHDyA was synthesized and purified as described earlier [28]. Briefly, hexadec-7-yn-1-ol was converted to hexadec-15-yn-1-ol via an alkyne zipper reaction, which was oxidized to hexadec-15-yn-1-al via Swern oxidation. Hexadec-15-yn-1-al was then subjected to organocatalytic α-chlorination to yield 2-ClHDyA.

### 4.11. CellTiter-Glo Assay

Briefly, HL-1 cells were seeded in 96 well plates and incubated in the presence of the indicated concentrations of 2-ClHDyA and 2-ClHDA (in DMSO). At the indicated time points, cell viability was measured using CellTiter-Glo^®^ 3D cell viability assay kit (Promega) according to the manufacturer’s recommendations. The CellTiter-Glo assay detects ATP as an indicator of cell viability [76].

### 4.12. Identification of 2-ClHDyA-Modified Proteins in HL-1 Cardiomyocytes

HL-1 cells were seeded in uncoated 6-well plates (to avoid binding of the aldehydes to the fibronectin/gelatine coating) and grown to confluence. Following pre-incubation in serum-free medium, cells were treated with 2-ClHDyA and 10-undecyn-1-ol (used as a negative control), both dissolved in DMSO at the indicated concentrations for the indicated times (37 °C). Schiff bases were reduced with NaCNBH_3_ (90 min at 37 °C). Cells were then washed with ice-cold PBS, lysed in clicking buffer (50 mM Tris/Cl, 1% SDS, pH 8.0), sonicated (twice for 5 s), and stored at −20 °C until further use.

### 4.13. Click Chemistry

Equal amounts of protein lysates of 2-ClHDyA and 10-undecyn-1-ol-treated HL-1 cells were subjected to click chemistry using the Click-it^®^ Protein Reaction Buffer Kit (Thermo, San Jose, CA, USA) in accordance to the manufacturer’s protocol and [32]. N_3_-TAMRA (in DMSO, 2 mM) was used as azide-containing detection reagent. Following click reaction precipitated proteins were stored at −20 °C until further use.

### 4.14. 1D-Sodium Dodecyl Sulfate-Polyacrylamide Gel Electrophoresis (SDS-PAGE)

To study the kinetics of the click reaction, 1D-SDS-PAGE analysis of TAMRA-labeled protein precipitates was performed. The final protein concentration was adjusted to 2 mg/mL. Samples were then heated for 10 min at 70 °C and 40 µg of protein were loaded. SDS-PAGE (12%) was carried out at 150 V. Gels were imaged on a Typhoon 9400 scanner (Amersham, ex 532 nm/em 580 nm). To normalize the fluorescence signal intensity on protein content/lane, Coomassie staining was performed and visualized on a ChemiDoc™ System (Biorad, Vienna, Austria).

### 4.15. 2D-GE

TAMRA-labelled protein precipitates (500 µg) were dissolved in 200 µL sample buffer, vortexed and incubated for 30 min at RT. To prevent adverse isoelectric of proteins, 300 µL of reswelling solution were added, samples were centrifuged and applied to IPG strips. Buffers and isoelectric focusing parameters are described in detail in [77]. Fluorescence imaging of 2D-gels was carried out on a Typhoon 9400 scanner. Spots were identified using the DeCyder Differential Analysis Software (Amersham Biosciences, Amersham, UK), picked manually and stored in H_2_O at −20 °C until in-gel tryptic digestion and extraction of peptides.

### 4.16. Coomassie-Blue Staining of 2D-gels

2D-gels with 10-undecyn-1-ol treated cell lysates, which served as a negative control to verify the sensitivity of the click-reaction, were subjected to Coomassie-blue staining for 2 h to reveal the overall protein pattern. Imaging was carried out on a ChemiDoc™ System.

### 4.17. Liquid Chromatography (LC)-MS/MS Analysis

LC-MS/MS analysis was performed as described earlier [77]. Dried peptide extracts were dissolved in 100 µL of 0.1% formic acid and analyzed on a nano-HPLC system (LC-20nano, Shimadzu; Vienna, Austria). Then, 50 µL samples were injected and concentrated on the loading column (LC Packings C18 Pep- Map™, 5 µm, 100 Å, 300 µm inner diameter × 1 mm) for 5 min using 0.1% formic acid as isocratic solvent at a flow rate of 20 µL/min. The column was then switched into the nanoflow circuit, and the sample was loaded on the nanocolumn (LC-Packings C18 PepMap™, 75 µm inner diameter × 150 mm) at a flow rate of 300 nL/min and separated using the following gradient: solvent A: water, 0.3% formic acid, solvent B: acetonitrile/water (80/20, *v*/*v*), 0.3% formic acid; 0 to 5 min: 4% B, after 40 min 55% B, then for 5 min 90% B and 47 min reequilibration at 4% B. The sample was ionized in a Finnigan nano-ESI source equipped with NanoSpray tips (PicoTip™ Emitter, New Objective, Woburn, MA, USA) and analyzed in a Thermo-Finnigan LTQ linear iontrap mass-spectrometer (Thermo, San Jose, CA, USA). MS/MS data were analyzed by searching the SwissProt public database with SpectrumMill Rev. B.04.01.141 (Agilent, Darmstadt, Germany) software. Acceptance was a protein score of >20 and individual peptide scores >7.5.

### 4.18. STRING Network Analysis

To visualize pathways and biological networks relevant to the identified proteins, STRING v10 [78] was used. The minimal required interaction score was set to medium confidence (0.400).

### 4.19. Statistical Analysis

All experiments were performed using three replicates per experimental group and repeated three times (unless otherwise stated). Statistical analyses were performed using the GraphPad Prism version 6 for Mac (GraphPad Software, Inc., San Diego, CA, USA). Data obtained from independent measurements were analyzed by Student‘s unpaired *t*-test and presented as mean ± SD or mean ± SEM (as indicated).

## Figures and Tables

**Table 1:** 2-ClHDyA-modified proteins identified in whole cell lysates of HL-1 cardiomyocytes. Only proteins identified in two out of three gels are displayed.

Uniprot ID	Short Name	Group	Full Name	Molecular Mass (Da)	Isoelectric Point (pI)
Q8BFZ3	Actbl2		Beta-actin-like protein 2	42,345.8	5.31
Q99KJ8	Dctn2		Dynactin subunit 2	44,230.2	5.14
P31001	Des		Desmin	53,553.9	5.21
P48678	Lmna		Prelamin-A/C	74,521.5	6.54
P15331	Prph		Peripherin	54,380.8	5.40
P14206	Rpsa		40S ribosomal protein SA	32,951.6	4.80
P05214	Tuba3b		Tubulin alpha-3 chain	50,643.3	4.98
Q3UX10	Tubal3		Tubulin alpha chain-like 3	50,728.9	5.37
Q9ERD7	Tubb3		Tubulin beta-3 chain	50,874.2	4.82
Q922F4	Tubb6		Tubulin beta-6 chain	50,545.9	4.80
P20152	Vim		Vimentin	53,743.8	5.06
Q9CQV8	Ywhab		14-3-3 protein beta/alpha	28,200.0	4.77
P80318	Cct3		T-complex protein 1 subunit gamma	61,199.5	6.28
P80316	Cct5		T-complex protein 1 subunit epsilon	60,079.5	5.72
P42932	Cct8		T-complex protein 1 subunit theta	60,125.0	5.44
P62737	Eif3f		Eukaryotic translation initiation factor 3 subunit F	38,097.7	5.33
P10630	Eif4a2		Eukaryotic initiation factor 4A-II	46,629.7	5.33
P30416	Fkbp4		Peptidyl-prolyl cis-trans isomerase FKBP4	51,970.8	5.54
P07901	Hsp90aa1		Heat shock protein HSP 90-alpha	85,185.7	4.93
P16627	Hspa1l		Heat shock 70 kDa protein 1-like	71,035.4	5.91
P14602	Hspb1		Heat shock protein beta-1	23,070.5	6.12
P27773	Pdia3		Protein disulfide-isomerase A3	57,133.7	5.88
Q922R8	Pdia6		Protein disulfide-isomerase A6	38,097.7	5.33
Q9WVJ2	Psmd13		26S proteasome non-ATPase regulatory subunit 13	43,150.9	5.46
Q60864	Stip1		Stress-induced-phosphoprotein 1	63,208.6	6.41
P11983	Tcp1		T-complex protein 1 subunit alpha	60,904.0	5.82
P10518	Alad		Delta-aminolevulinic acid dehydratase	36,479.3	6.32
P47738	Aldh2		Aldehyde dehydrogenase, mitochondrial	57,050.0	7.76
P05064	Aldoa		Fructose-bisphosphate aldolase A	39,811.6	8.75
P17182	Eno1		Alpha-enolase	47,482.3	6.37
P16858	Gapdh		Glyceraldehyde-3-phosphate dehydrogenase	36,094.6	8.76
Q9D6R2	Idh3a		Isocitrate dehydrogenase [NAD] subunit alpha, mitochondrial	40,094.5	6.27
P06151	Ldha		L-lactate dehydrogenase A chain	36,840.2	7.84
Q91WD5	Ndufs2		NADH dehydrogenase [ubiquinone] iron-sulfur protein 2, mitochondrial	53,024.1	6.52
Q9DBJ1	Pgam1		Phosphoglycerate mutase 1	28,945.6	6.68
Q61753	Phgdh		D-3-phosphoglycerate dehydrogenase	57,383.3	6.12
Q9CZ13	Uqcrc1		Cytochrome b-c1 complex subunit 1, mitochondrial	53,478.4	5.81
Q9DB77	Uqcrc2		Cytochrome b-c1 complex subunit 2, mitochondrial	48,291.1	9.31
P07724	Alb		Albumin	70,745.2	5.75
Q9Z1Q5	Clic1		Chloride intracellular channel protein 1	27,354.6	5.09
Q8VDW0	Ddx39		ATP-dependent RNA helicase DDX39A	49,580.0	5.46
Q9Z1N5	Ddx39b		Spliceosome RNA helicase Ddx39b	49,491.0	5.44
Q9D8N0	Eef1g		Elongation factor 1-gamma	50,402.1	6.31
P58252	Eef2		Elongation factor 2	96,282.3	6.41
Q920E5	Fdps		Farnesyl pyrophosphate synthase	40,923.1	5.49
Q8R081	Hnrnpl		Heterogeneous nuclear ribonucleoprotein L	64,590.1	8.75
P17897	Lyz1		Lysozyme C-1	17,250.3	10.35
P17918	Pcna		Proliferating cell nuclear antigen	29,126.7	4.66
Q91VI7	Rnh1		Ribonuclease inhibitor	51,527.2	4.69
Q9WTM5	Ruvbl2		RuvB-like 2	51,282.8	5.49
Q3TW96	Uap1l1		UDP-N-acetylhexosamine pyrophosphorylase-like protein 1	57,354.4	5.27

Blue group: Cytoskeleton; Red Group: Chaperones & Stress Response; Green group: Energy Metabolism; Grey Group: Miscellaneous.

**Table 2:** Characteristic *m/z* values used to identify and quantify chlorinated analytes.

Analyte (PFB-Derivatives)	*m/z*	Internal Standard (I.S.)	*m/z*	I.S. (ng)
**2-ClHDA**	288/290	2-Cl[^13^C_8_]HDA	296/298	1000
**2-ClHA**	289/291	2-Cl[^13^C_8_]HA	297/299	1000
**2-ClHOH**	470/472	pentadecanol	422	1000

## Supplementary Materials

Supplementary materials can be found at https://www.mdpi.com/1422-0067/21/23/9235/s1.

## Abbreviations

2-ClHA	2-chlorohexadecanoic acid
2-ClHDA	2-chlorohexadecanal
2-ClHDyA	2-chlorohexadec-15-yn-1-al
2-ClHOH	2-chlorohexadecanol
2D-GE	Two dimensional-gel electrophoresis
CCT	Chaperonin-containing T-complex
DMSO	Dimethyl sulfoxide
FA	Fatty acid
FALDH	Fatty aldehyde dehydrogenase
FBS	Fetal bovine serum
GO	Gene ontology
GSH	Glutathione
HNE	4-Hydroxynonenal
HOCl	Hypochlorous acid
HRP	Horseradish peroxidase
HSP	Heat shock protein
HSR	Heat shock response
LC-MS/MS	Liquid chromatography-tandem mass spectrometry
LPS	Lipopolysaccharide
MI	Myocardial infarction
MPO	Myeloperoxidase
N_3_-TAMRA	5-tetramethylrhodamin azide
NETs	Neutrophil extracellular traps
NICI-GC-MS	Negative ion chemical ionization-gas chromatography-mass spectrometry
NO	Nitric oxide
PBS	Phosphate-buffered saline
PFB	Pentafluorobenzyl
RES	Reactive electrophile species
ROS	Reactive oxygen species
RT	Room temperature
SDS-PAGE	Sodium dodecyl sulfate-polyacrylamide gel electrophoresis
SIM	Selected ion monitoring
TLR4	Toll-like receptor 4
TNFα	Tumor necrosis factor α
