# Supplementary material for: Myeloperoxidase-Derived 2-Chlorohexadecanal Is Generated in Mouse Heart during Endotoxemia and Induces Modification of Distinct Cardiomyocyte Protein Subsets In Vitro
Source: Int J Mol Sci. 2020 Dec 3;21(23):9235. doi: 10.3390/ijms21239235 (PMC7730634; doi:10.3390/ijms21239235)
Supplement: Supplementary file 1 [file ijms-21-09235-s001.pdf]

# SUPPLEMENTARY INFORMATION

## Myeloperoxidase-derived 2-chlorohexadecanal is generated in mouse heart during endotoxemia and induces modification of distinct cardiomyocyte protein subsets in vitro

Jürgen Prasch, Eva Bernhart, Helga Reicher, Manfred Kollroser, Gerald N. Rechberger, Chintan N. Koyani, Christopher Trummer, Lavinia Rech, Peter P. Rainer, Astrid Hammer, Ernst Malle, Wolfgang Sattler

**Table S1:** Biological process gene ontology (GO) enrichment analysis.

| #term ID | term description | observed gene count | background gene count | false discovery rate | matching proteins in network (labels) |
|---|---|---|---|---|---|
| GO:0006457 | protein folding | 10 | 153 | 5.21e-09 | Cct3,Cct5,Cct8,Fkbp4,Hsp90aa1,Hspa1l,Hspb1,Pdia3,Pdia6,Tcp1 |
| GO:0007339 | binding of sperm to zona pellucida | 6 | 36 | 4.02e-07 | Aldoa,Cct3,Cct5,Cct8,Hspa1l,Tcp1 |
| GO:0061077 | chaperone-mediated protein folding | 6 | 60 | 2.67e-06 | Cct3,Cct5,Cct8,Fkbp4,Hspb1,Tcp1 |
| GO:0017144 | drug metabolic process | 11 | 494 | 4.06e-06 | Aldh2,Aldoa,Eno1,Gapdh,Hsp90aa1,Idh3a,Ldha,Ndufs2,Pgam1,Phgdh,Uqcrc1 |
| GO:2000573 | positive regulation of DNA biosynthetic process | 6 | 69 | 4.16e-06 | Cct3,Cct5,Cct8,Ddx39b,Hsp90aa1,Tcp1 |
| GO:0009987 | cellular process | 47 | 12459 | 4.22e-06 | Alad,Alb,Aldh2,Aldoa,Cct3,Cct5,Cct8,Dctn2,Ddx39,Ddx39b,Des,Eef1g,Eef2,Eif3f,Eif4a2,Eno1,Fdps,Fkbp4,Gapdh,Hnrnpl,Hsp90aa1,Hspa1l,Hspb1,Idh3a,Ldha,Lmna,Lyz1,Ndufs2,Pcna,Pdia3,Pdia6,Pgam1,Phgdh,Prph,Psmd13,Rpsa,Ruvbl2,Tcp1,Tuba3b,Tubal3,Tubb3,Tubb6,Uap1l1,Uqcrc1,Uqcrc2,Vim,Ywhab |
| GO:1904851 | positive regulation of establishment of protein localization to telomere | 4 | 10 | 4.22e-06 | Cct3,Cct5,Cct8,Tcp1 |
| GO:0046031 | ADP metabolic process | 5 | 44 | 1.07e-05 | Aldoa,Eno1,Gapdh,Ldha,Pgam1 |
| GO:0046034 | ATP metabolic process | 7 | 162 | 1.15e-05 | Aldoa,Eno1,Gapdh,Ldha,Ndufs2,Pgam1,Uqcrc1 |
| GO:0046496 | nicotinamide nucleotide metabolic process | 6 | 101 | 1.55e-05 | Aldoa,Eno1,Gapdh,Idh3a,Ldha,Pgam1 |
| GO:0006090 | pyruvate metabolic process | 5 | 62 | 2.34e-05 | Aldoa,Eno1,Gapdh,Ldha,Pgam1 |
| GO:0006091 | generation of precursor metabolites and energy | 8 | 295 | 2.34e-05 | Aldoa,Eno1,Gapdh,Idh3a,Ldha,Ndufs2,Pgam1,Uqcrc1 |
| GO:0009167 | purine ribonucleoside monophosphate metabolic process | 7 | 196 | 2.34e-05 | Aldoa,Eno1,Gapdh,Ldha,Ndufs2,Pgam1,Uqcrc1 |
| GO:0051054 | positive regulation of DNA metabolic process | 7 | 223 | 3.96e-05 | Cct3,Cct5,Cct8,Ddx39b,Hsp90aa1,Pcna,Tcp1 |
| GO:0032212 | positive regulation of telomere maintenance via telomerase | 4 | 31 | 4.95e-05 | Cct3,Cct5,Cct8,Tcp1 |
| GO:0032204 | regulation of telomere maintenance | 5 | 78 | 5.10e-05 | Cct3,Cct5,Cct8,Lmna,Tcp1 |
| GO:0016052 | carbohydrate catabolic process | 5 | 80 | 5.59e-05 | Aldoa,Eno1,Gapdh,Ldha,Pgam1 |
| GO:0006458 | 'de novo' protein folding | 4 | 35 | 6.80e-05 | Cct3,Cct5,Cct8,Tcp1 |
| GO:0050821 | protein stabilization | 6 | 157 | 6.85e-05 | Cct3,Cct5,Cct8,Gapdh,Hsp90aa1,Tcp1 |
| GO:0006096 | glycolytic process | 4 | 37 | 7.79e-05 | Aldoa,Eno1,Gapdh,Pgam1 |
| GO:0006757 | ATP generation from ADP | 4 | 37 | 7.79e-05 | Aldoa,Eno1,Gapdh,Pgam1 |
| GO:0009166 | nucleotide catabolic process | 5 | 90 | 8.27e-05 | Aldoa,Eno1,Gapdh,Ldha,Pgam1 |
| GO:0051052 | regulation of DNA metabolic process | 8 | 383 | 8.94e-05 | Cct3,Cct5,Cct8,Ddx39b,Hsp90aa1,Lmna,Pcna,Tcp1 |
| GO:0042866 | pyruvate biosynthetic process | 4 | 41 | 0.00010 | Aldoa,Eno1,Gapdh,Pgam1 |
| GO:1901998 | toxin transport | 4 | 42 | 0.00011 | Cct3,Cct5,Cct8,Tcp1 |
| GO:0051186 | cofactor metabolic process | 8 | 403 | 0.00012 | Alad,Aldoa,Eef1g,Eno1,Gapdh,Idh3a,Ldha,Pgam1 |
| GO:0055086 | nucleobase-containing small molecule metabolic process | 9 | 566 | 0.00016 | Aldoa,Eno1,Gapdh,Idh3a,Ldha,Ndufs2,Pgam1,Uap1l1,Uqcrc1 |
| GO:0019359 | nicotinamide nucleotide biosynthetic process | 4 | 54 | 0.00025 | Aldoa,Eno1,Gapdh,Pgam1 |
| GO:0009117 | nucleotide metabolic process | 8 | 488 | 0.00039 | Aldoa,Eno1,Gapdh,Idh3a,Ldha,Ndufs2,Pgam1,Uqcrc1 |
| GO:0016043 | cellular component organization | 24 | 4560 | 0.00051 | Alad,Aldoa,Cct3,Cct8,Dctn2,Ddx39b,Des,Fkbp4,Gapdh,Hsp90aa1,Lmna,Pcna,Phgdh,Prph,Psmd13,Rpsa,Ruvbl2,Tuba3b,Tubal3,Tubb3,Tubb6,Uqcrc |

| | | | | | |
|---|---|---|---|---|---|
| | | | | | 2,Vim,Ywhab |
| GO:0006754 | ATP biosynthetic process | 4 | 70 | 0.00054 | Aldoa,Eno1,Gapdh,Pgam1 |
| GO:0046394 | carboxylic acid biosynthetic process | 6 | 256 | 0.00061 | Aldoa,Eno1,Gapdh,Ldha,Pgam1,Phgdh |
| GO:0055114 | oxidation-reduction process | 10 | 898 | 0.00069 | Aldh2,Gapdh,Idh3a,Ldha,Ndufs2,Pdia3,Pdia6,Phgdh,Uqcrc1,Uqcrc2 |
| GO:0051701 | interaction with host | 4 | 78 | 0.00073 | Alb,Ddx39b,Gapdh,Tcp1 |
| GO:0007010 | cytoskeleton organization | 10 | 916 | 0.00080 | Dctn2,Des,Gapdh,Lmna,Prph,Tuba3b,Tubal3,Tubb3,Tubb6,Vim |
| GO:0072330 | monocarboxylic acid biosynthetic process | 5 | 164 | 0.00080 | Aldoa,Eno1,Gapdh,Ldha,Pgam1 |
| GO:1901566 | organonitrogen compound biosynthetic process | 11 | 1122 | 0.00080 | Alad,Aldoa,Eef1g,Eef2,Eif3f,Eif4a2,Eno1,Gapdh,Pgam1,Phgdh,Rpsa |
| GO:2001252 | positive regulation of chromosome organization | 5 | 166 | 0.00080 | Cct3,Cct5,Cct8,Ruvbl2,Tcp1 |
| GO:0051704 | multi-organism process | 14 | 1840 | 0.00091 | Alad,Alb,Aldoa,Cct3,Cct5,Cct8,Ddx39b,Eif3f,Fkbp4,Gapdh,Hspa1l,Hspb1,Lyz1,Tcp1 |
| GO:1904951 | positive regulation of establishment of protein localization | 7 | 424 | 0.00094 | Cct3,Cct5,Cct8,Gapdh,Hsp90aa1,Hspa1l,Tcp1 |
| GO:0051188 | cofactor biosynthetic process | 5 | 175 | 0.00097 | Alad,Aldoa,Eno1,Gapdh,Pgam1 |

**Table S2:** KEGG pathway analysis.

| #term ID | term description | observed gene count | background gene count | false discovery rate | matching proteins in network (labels) |
|---|---|---|---|---|---|
| mmu00010 | Glycolysis / Gluconeogenesis | 6 | 65 | 1.25e-06 | Aldh2,Aldoa,Eno1,Gapdh,Ldha,Pgam1 |
| mmu01230 | Biosynthesis of amino acids | 6 | 75 | 1.40e-06 | Aldoa,Eno1,Gapdh,Idh3a,Pgam1,Phgdh |
| mmu01200 | Carbon metabolism | 6 | 118 | 1.19e-05 | Aldoa,Eno1,Gapdh,Idh3a,Pgam1,Phgdh |
| mmu01100 | Metabolic pathways | 14 | 1296 | 2.21e-05 | Alad,Aldh2,Aldoa,Eno1,Fdps,Gapdh,Idh3a,Ldha,Ndufs2,Pgam1,Phgdh,Uap1l1,Uqcrc1,Uqcrc2 |
